# Neuropathic Pain Associated With First Metatarsophalangeal Joint Osteoarthritis: Frequency and Associated Factors

**DOI:** 10.1002/acr.25125

**Published:** 2023-05-11

**Authors:** Hylton B. Menz, Jamie J. Allan, Andrew K. Buldt, Karl B. Landorf, Flavia M. Cicuttini, Edward Roddy, Shannon E. Munteanu

**Affiliations:** ^1^ La Trobe University Melbourne Victoria Australia; ^2^ Monash University and Alfred Hospital Melbourne Victoria Australia; ^3^ Keele University, Keele, Staffordshire, UK, and Haywood Hospital Burslem Staffordshire UK

## Abstract

**Objective:**

To determine whether neuropathic pain is a feature of first metatarsophalangeal (MTP) joint osteoarthritis (OA).

**Methods:**

A total of 98 participants (mean ± SD age 57.4 ± 10.3 years) with symptomatic radiographic first MTP joint OA completed the PainDETECT questionnaire (PD‐Q), which has 9 questions regarding the intensity and quality of pain. The likelihood of neuropathic pain was determined using established PD‐Q cutoff points. Participants with unlikely neuropathic pain were then compared to those with possible/likely neuropathic pain in relation to age, sex, general health (Short Form 12 [SF‐12] health survey), psychological well‐being (Depression, Anxiety and Stress Scale), pain characteristics (self‐efficacy, duration, and severity), foot health (Foot Health Status Questionnaire [FHSQ]), first MTP dorsiflexion range of motion, and radiographic severity. Effect sizes (Cohen's *d* coefficient) were also calculated.

**Results:**

A total of 30 (31%) participants had possible/likely neuropathic pain (19 possible [19.4%], 11 likely [11.2%]). The most common neuropathic symptoms were sensitivity to pressure (56%), sudden pain attacks/electric shocks (36%) and burning (24%). Compared to those with unlikely neuropathic pain, those with possible/likely neuropathic pain were significantly older (*d* = 0.59, *P* = 0.010), had worse SF‐12 physical scores (*d* = 1.10, *P* < 0.001), pain self‐efficacy scores (*d* = 0.98, *P* < 0.001), FHSQ pain scores (*d* = 0.98, *P* < 0.001), and FHSQ function scores (*d* = 0.82, *P* < 0.001), and had higher pain severity at rest (*d* = 1.01, *P* < 0.001).

**Conclusion:**

A significant proportion of individuals with first MTP joint OA report symptoms suggestive of neuropathic pain, which may partly explain the suboptimal responses to commonly used treatments for this condition. Screening for neuropathic pain may be useful in the selection of targeted interventions and may improve clinical outcomes.

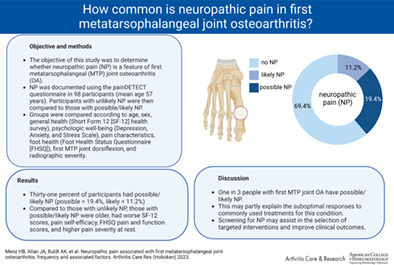

## INTRODUCTION

Pain is the most common and disabling symptom of osteoarthritis (OA) and has primarily been attributed to local tissue damage leading to mechanical and/or inflammatory stimulation of peripheral sensory neurons (nociceptors) in joint tissue (1). However, the suboptimal and variable response to treatment of OA‐related pain has led to reappraisal of its underlying cause, and the contribution of non‐nociceptive pathways is increasingly recognized (2, 3). In particular, neuropathic pain, defined as pain arising as a direct consequence of a lesion or disease affecting the somatosensory system (4), may be responsible for symptoms such as tingling, numbness, burning, and electrical shock sensations (5), which are experienced by one‐third of individuals with knee or hip OA (6).

**SIGNIFICANCE & INNOVATIONS**

- This is the first study to evaluate neuropathic pain in individuals with foot osteoarthritis (OA).
- One in 3 individuals with first metatarsophalangeal joint OA had evidence of possible or likely neuropathic pain.
- Those with neuropathic pain were older, had worse general physical health, worse foot health, and greater pain severity at rest.
- Screening for neuropathic pain may be useful in the selection of appropriate interventions.

The presence of neuropathic symptoms increases the individual burden of knee OA, as it is associated with more severe pain (7, 8, 9, 10), greater impairment in physical function (9, 10, 11, 12, 13), worse quality of life (10, 11, 13, 14), and poorer sleep quality (10). Several person‐level factors are associated with neuropathic pain in individuals with knee OA, including increased age (13), higher body mass index (BMI) (13), female sex (8), multiple comorbidities (8), pain at multiple sites (7, 12), referred pain (7), and hyperalgesia (9). Knee joint–specific correlations with neuropathic pain include meniscal lesions (15) and prior surgery (10), although reported associations with radiographic severity are inconsistent (12, 13, 16).

To the best of our knowledge, no studies have examined neuropathic pain related to OA affecting the joints of the foot. This is important since foot OA has a similar prevalence compared to knee OA (17), is considered disabling in 75% of patients (17), and is a common reason for consultation in primary care (18). Foot OA most commonly affects the first metatarsophalangeal (MTP) joint and is characterized by the formation of a dorsal exostosis (19), limited range of motion (20), and altered walking patterns (21). Interventions such as footwear and orthoses have been shown to alter the biomechanical function of the foot in individuals with first MTP joint OA (22, 23) but show only modest reductions in pain (24, 25), suggesting that non‐mechanical factors may contribute to symptoms.

Therefore, the objectives of this study were to determine whether neuropathic pain is a feature of first MTP joint OA and to explore person‐ and foot‐level factors associated with the presence of neuropathic pain in participants enrolled in a recent randomized clinical trial.

## PATIENTS AND METHODS

### Participants

Participants for this study were recruited from a randomized trial that evaluated the effectiveness of shoe‐stiffening inserts for first MTP joint OA. Full details of the trial have been described previously (25, 26). Participants were recruited using advertisements in local newspapers, posters placed in senior citizens’ centers and retirement villages, mailed advertisements to health care practitioners, mailed advertisements to individuals currently accessing podiatry services at the La Trobe University Health Sciences Clinic, and through social media. To be included in the trial, participants needed to be ≥18 years old, have pain in the first MTP joint on most days for at least 12 weeks, rated ≥30 mm on a 100‐mm visual analog scale, have pain upon palpation of the dorsal aspect of the first MTP joint, have restricted first MTP joint dorsiflexion, and be able to walk household distances without the use of a walking aid. Participants were excluded if they had previous first MTP joint surgery, were currently pregnant, or had hallux valgus, a systemic inflammatory condition, or cognitive impairment.

Ethical approval was provided by the La Trobe University Human Ethics Committee (approval no. HEC15‐128), and written informed consent was obtained from all participants. In this study, the sample size was determined by the requirements of the randomized trial, which was powered to detect a minimum clinically important difference in the primary outcome measure, the Foot Health Status Questionnaire (FHSQ) pain subscale (25, 26).

### Demographic, general health, and pain assessments

A structured questionnaire was used to collect data regarding participant demographic characteristics (age and sex), general health (the Short Form 12 questionnaire [27]), psychological well‐being (the Depression, Anxiety and Stress Scale [28]), pain characteristics (including the Pain Self‐Efficacy Questionnaire [PSEQ] [29], pain duration, and pain severity at rest and while walking [26]), and foot health (the FHSQ pain and function subscales [30]). Only baseline data were used in this analysis.

### Clinical and radiographic assessments

Height and weight were measured using a stadiometer and digital scales, and BMI was calculated as weight/height (kg/m^2^). Clinical features associated with first MTP joint OA (pain on palpation, a dorsal exostosis, joint effusion, pain on motion, hard end‐feel, and crepitus) and passive, non–weight‐bearing first MTP joint dorsiflexion range of motion were documented using established techniques (19). The presence of radiographic first MTP joint OA was determined using the La Trobe University Radiographic Atlas, which uses weight‐bearing dorsiplantar and lateral radiographs to document the presence of OA based on the observation of osteophytes and joint space narrowing (JSN) (31). Radiographic OA was documented as present or absent based on the La Trobe University Radiographic Atlas case definition (at least one score of 2 for osteophytes or JSN on either the dorsiplantar or lateral view) (32), and radiographic OA severity was documented as mild (no scores for osteophytes or JSN on either view >1), moderate (at least one score of 2 but none >2), or severe (at least one score of 3) (20).

### Neuropathic pain assessment

To document the presence of neuropathic pain affecting the first MTP joint, we used the self‐reported PainDETECT Questionnaire (PD‐Q), which was originally developed to discriminate between nociceptive pain and neuropathic pain in individuals with chronic low back pain (33). The PD‐Q comprises 7 items evaluating pain quality (scores from 0 to 5), 1 item evaluating pain pattern (scores from –1 to 1), and 1 item evaluating pain radiation (scores from 0 to 2). The sum of individual question scores was used to calculate a total score ranging from –1 to 38. Total scores <13 indicate that neuropathic pain is unlikely, scores from 13 to 18 indicate that neuropathic pain is possible, and scores >18 indicate that neuropathic pain is likely (34). The PD‐Q has been validated against expert physician diagnosis of neuropathic pain in individuals with low back pain (33) and against quantitative sensory testing for the detection of central sensitization in individuals with knee OA (35).

### Statistical analysis

Statistical analysis was undertaken using IBM SPSS Statistics version 26.0. All data were explored for normality and did not require transformation. For continuously scored variables, differences between participants with and those without neuropathic pain were compared using independent samples *t*‐tests and effect sizes (Cohen's *d* coefficient). The following interpretation of effect sizes was used: ≤0.01 indicates very small, >0.01 to 0.20 indicates small, >0.20 to 0.50 indicates medium, >0.50 to 0.8 indicates large, >0.80 to 1.2 indicates very large, and >1.20 indicates huge (36). For dichotomous or ordinal variables, differences between groups were calculated using the chi‐square statistic.

## RESULTS

### Participants

A total of 100 participants were recruited for the randomized trial (25). Of these, 98 participants had complete PD‐Q data and were included in this analysis (44 men and 54 women, mean ± SD age 57.3 ± 10.3 years). Participant characteristics are shown in Table 1. Data were missing for the following variables: height, weight, and BMI (n = 3), dorsiplantar radiographs (n = 5), and lateral radiographs (n = 6).

**Table 1:** Demographic and clinical characteristics of 98 participants with radiographic first MTP joint OA*

Characteristics	Values
Demographic characteristics and anthropometrics	
Age, mean ± SD years	57.3 ± 10.3
Female sex	54 (55.1)
Height, mean ± SD cm	168.3 ± 8.2
Weight, mean ± SD kg	79.4 ± 13.0
BMI, mean ± SD kg/m^2^	28.1 ± 4.6
Clinical features	
Passive, non–weight‐bearing first MTP joint maximum dorsiflexion, mean ± SD degrees	45.3 ± 11.2
Pain on palpation	98 (100.0)
Palpable dorsal exostosis	97 (99.0)
Pain on motion of first MTP joint	74 (75.5)
Hard end‐feel when dorsiflexed	92 (93.9)
Crepitus	21 (21.4)
Radiographic first MTP joint OA†	84 (90.3)
Radiographic severity‡	
Mild	9 (9.7)
Moderate	38 (40.9)
Severe	46 (49.5)

* Except where indicated otherwise, values are the number (%). MTP = metatarsophalangeal; OA = osteoarthritis.

† Indicates at least one score of 2 for osteophytes or joint space narrowing on either view using the case definition from the La Trobe Radiographic Atlas (31).

‡ Mild indicates no scores >1; moderate indicates at least one score of 2 but none >2; severe: at least one score of 3 for osteophytes or joint space narrowing on either view, using the La Trobe Radiographic Atlas (31).

### Neuropathic pain characteristics

PD‐Q responses are shown in Table 2. A total of 69 of the 98 participants (70%) reported at least 1 neuropathic symptom with at least moderate severity, with the most common neuropathic symptoms being sensitivity to pressure (n = 55 [56%]), sudden pain attacks/electric shocks (n = 35 [36%]), and burning (n = 24 [25%]). A total of 37 participants (37.8%) reported pain radiation. Thirty (31%) participants had possible/likely neuropathic pain (n = 19 [19.4%], n = 11 [11.2%], for possible neuropathic pain and likely neuropathic pain, respectively), as defined according to overall PD‐Q score.

**Table 2:** PainDETECT responses in 98 participants with radiographic first MTP joint OA*

Characteristics	Values
Pain severity, mean ± SD (score 0–10)	
How would you assess your pain now, at this moment?	3.76 ± 2.34
How strong was the strongest pain during the past 4 weeks?	7.03 ± 2.02
How strong was the pain during the past 4 weeks on average?	4.96 ± 1.86
Pain pattern	
Persistent pain with slight variations	32 (32.7)
Persistent pain with pain attacks	33 (33.7)
Pain attacks without pain between them	25 (25.5)
Pain attacks with pain between them	8 (8.2)
Pain radiation	37 (37.8)
Pain quality, moderate or more (score ≥3 [of 5])	
Burning	24 (24.5)
Tingling or prickling	14 (14.3)
Sensitivity to light touch	18 (18.4)
Sudden pain attacks/electric shocks	35 (35.7)
Sensitivity to cold or heat	10 (10.2)
Numbness	12 (12.2)
Sensitivity to pressure	55 (56.1)
Total PainDETECT score, mean ± SD (score 0–38)†	10.7 ± 5.5
Neuropathic pain unlikely	68 (69.4)
Neuropathic pain possible	19 (19.4)
Neuropathic pain likely	11 (11.2)

* Except where indicated otherwise, values are the number (%) of participants. See Table 1 for definitions.

† Total scores <13 indicate that neuropathic pain is unlikely; scores of 13–18 indicate that neuropathic pain is possible; and scores >18 indicate that neuropathic pain is likely (34).

### Differences between participants with and those without neuropathic pain

Participant characteristics in those with and those without neuropathic pain are shown in Table 3. Compared to those with unlikely neuropathic pain, those with possible/likely neuropathic pain were significantly older (*d* = 0.59, *P* = 0.010; large effect size), had worse scores on the questionnaires for SF‐12 physical function (*d* = 1.10, *P* < 0.001; very large effect size), PSEQ (*d* = 0.98, *P* < 0.001; very large effect size), FHSQ pain (*d* = 0.98, *P* < 0.001; very large effect size), and FHSQ function (*d* = 0.82, *P* < 0.001; very large effect size), and had higher pain severity at rest (*d* = 1.01, *P* < 0.001; very large effect size).

**Table 3:** Demographic and clinical characteristics in OA participants with and those without neuropathic pain affecting the first MTP joint*

Characteristics	Non‐neuropathic (n = 68)	Neuropathic (n = 30)	*d*	*P*
Demographic characteristics and anthropometrics				
Age, mean ± SD years	55.5 ± 11.0	61.3 ± 7.1	0.59	0.003
Female sex	34 (50.0)	20 (66.7)	–	0.186
BMI, mean ± SD kg/m^2^	27.6 ± 4.6	29.3 ± 4.5	0.38	0.092
General health (SF‐12 scores)†				
Physical	49.2 ± 8.0	39.9 ± 9.7	1.10	<0.001
Mental	53.7 ± 9.4	52.5 ± 8.7	0.13	0.543
Psychological well‐being (DASS‐21)‡				
Depression	2.9 ± 5.9	4.9 ± 5.7	0.35	0.118
Anxiety	3.2 ± 5.3	3.6 ± 4.9	0.08	0.723
Stress	7.3 ± 7.4	9.3 ± 8.8	0.26	0.287
Pain characteristics				
PSEQ§	54.1 ± 6.0	47.0 ± 9.7	0.98	0.001
Pain duration, months	39 ± 47	60 ± 92	0.35	0.055
Pain severity at rest, VAS¶	2.4 ± 1.6	4.1 ± 1.9	1.01	<0.001
Pain severity while walking, VAS¶	5.0 ± 1.5	5.5 ± 1.6	0.33	0.164
Foot health (FHSQ scores)#				
Pain	51.9 ± 16.1	37.0 ± 13.4	0.98	<0.001
Function	71.7 ± 21.6	53.8 ± 23.1	0.82	<0.001
Clinical features				
Passive non–weight‐bearing first MTP joint maximum dorsiflexion, mean ± SD degrees	46.6 ± 10.1	42.2 ± 13.1	0.40	0.108
Pain on palpation	68 (100.0)	30 (100.0)	–	NC
Palpable dorsal exostosis	67 (98.5)	30 (100.0)	–	0.504
Pain on motion of first MTP joint	48 (70.6)	26 (86.7)	–	0.088
Hard end‐feel when dorsiflexed	64 (94.1)	28 (93.3)	–	0.881
Crepitus	14 (20.6)	7 (23.3)	–	0.760
Radiographic first MTP joint OA**	59 (90.8)	25 (89.3)	–	0.546
Radiographic severity††				
Mild	6 (9.2)	3 (10.7)	–	0.965
Moderate	27 (41.5)	11 (39.3)	–	–
Severe	32 (49.2)	14 (50.0)	–	–

* Except where indicated otherwise, values are the number (%) of participants. MTP = metatarsophalangeal; NC = not calculable; OA = osteoarthritis.

† For short Form 12 (SF‐12) scores, scores ranged from 0 to 100, with higher scores indicating better function.

‡ For 21‐item Depression, Anxiety and Stress Scale (DASS‐21) scores, scores ranged from 0 to 42, with higher scores indicating worse function.

§ For Pain Self‐Efficacy (PSEQ) questionnaire scores, score ranged from 0 to 60, with higher scores indicating greater confidence dealing with pain.

¶ For visual analog scale (VAS) scores, score ranged from 0 to 10, with higher scores indicating worse pain.

# For Foot Health Status Questionnaire (FHSQ)scores, score ranged from 0 to100, with higher scores indicating better function.

** At least one score of 2 for osteophytes or joint space narrowing on either view using the case definition from the La Trobe Radiographic Atlas (31).

†† Mild indicates no scores >1; moderate indicates at least one score of 2 but none >2; severe indicates at least one score of 3 for osteophytes or joint space narrowing on either view, using the La Trobe Radiographic Atlas (31).

## DISCUSSION

In this study, we aimed to determine whether neuropathic pain is a feature of foot OA by using the PD‐Q in OA participants with first MTP joint OA who were enrolled in a randomized trial. We found that 70% of participants reported ≥1 moderate symptom indicative of neuropathic pain (such as electric shocks, burning, numbness, and tingling), and that the prevalence of possible/likely neuropathic pain in this group using the established overall PD‐Q cutoff score was 31%. Those with possible/likely neuropathic pain were older, had worse general physical health, worse foot health, and greater pain severity at rest. To the best of our knowledge, this study provides the first insights into neuropathic pain related to foot OA.

The prevalence of neuropathic pain observed in this study is similar to previous reports in individuals with knee and hip OA. A systematic review and meta‐analysis of 39 studies (36 involving the knee and 3 involving the hip) showed a pooled prevalence estimate of 40% (95% confidence interval [95% CI] 32–48) in knee OA and 29% (95% CI 22–37) in hip OA, using the same case definition of possible/likely neuropathic pain from the PD‐Q (6). The prevalence of reporting individual neuropathic symptoms was also high in our study, with 70% reporting ≥1 neuropathic symptom with at least moderate severity. The most frequently reported symptoms—sensitivity to pressure and sudden electric shocks—are hallmark features of neuropathic pain and are believed to result from central sensitization and spontaneous firing of peripheral nociceptors, respectively (37).

We observed several person‐level but few foot‐level differences between participants with and those without neuropathic pain. Those with neuropathic pain had worse general health (demonstrated by lower SF‐12 scores) and greater pain severity, which is consistent with previous reports related to neuropathic pain in individuals with knee OA using a range of health‐related quality of life measures (11, 13, 14) and pain assessment tools (7, 8, 10, 15). Interestingly, we found that although pain severity at rest was higher in those with neuropathic pain, pain during walking was not. This provides further evidence of a centrally mediated pain process in some participants, since pain severity when walking is typically greater than at rest in first MTP joint OA (25), possibly due to the loads associated with walking leading to mechanical stimulation of sensory neurons in local joint tissue.

The contribution of local, joint‐level factors to neuropathic pain in OA is unclear. Although neuropathic pain in individuals with knee OA is more common in those with meniscal lesions (15) or those who have undergone surgery (10), findings related to the association with radiographic severity are inconsistent (12, 13, 16) and may be confounded by the influence of disease duration. We found no difference between the non‐neuropathic group and neuropathic group in relation to measures of disease severity, including clinical features (such as range of motion, crepitus, or presence of a dorsal exostosis) or the presence and severity of radiographic OA. This is a notable finding, since previous studies demonstrated several dose‐response relationships between radiographic severity of first MTP joint OA, range of motion, and symptoms, consistent with a longitudinal pattern of progression (20).

Taken together, these findings suggest that while local structural factors may play a role in first MTP joint OA disease progression and symptoms more broadly, neuropathic symptoms may be more closely related to systematic factors. However, it is also possible that the initial catalyst for OA symptoms is mechanical, and prolonged nociceptive input subsequently leads to neuropathic pain symptoms via central sensitization (5). Although the relationship was not statistically significant (*P* = 0.055), participants in our study with possible/likely neuropathic pain had a longer duration of symptoms (mean of 60 months versus 39 months).

The key clinical implication of these findings is that there may be some value in screening for neuropathic symptoms in individuals with first MTP joint OA, as this may influence treatment decisions. Emerging evidence suggests that individuals with neuropathic pain associated with knee OA may be less responsive to commonly used treatments such as physical therapy (38) or joint replacement surgery (39). Although no studies have explored this in relation to foot OA, the presence of neuropathic pain may at least partly explain why only modest improvements of symptoms have been observed in clinical trials of footwear and foot orthoses, interventions that address mechanical deficits associated with first MTP joint OA (24, 25, 40). In individuals with predominantly neuropathic symptoms, centrally acting pharmacologic treatment approaches may be indicated (2), although only duloxetine, a serotonin–norepinephrine reuptake inhibitor, has sufficient evidence to support its use in OA (41).

Strengths of this study include the well‐characterized sample with validated clinical and radiographic measures of first MTP joint OA and a broad array of general health measures. However, our findings need to be considered in the context of several inherent limitations of the study design. First, our participants were recruited from a randomized trial rather than a population‐based cohort, so the sample size was relatively small and may not be reflective of the broader population with first MTP joint OA. Second, our case definition for neuropathic pain was based on the PD‐Q. Although this is a commonly used tool with some evidence of validity, there is currently no gold standard to definitively identify OA‐associated neuropathic pain. We also used the original PD‐Q rather than the modified version, the latter of which may have better validity, since it requests participants focus on neuropathic symptoms in or around the affected joint rather than their main area of pain, and the pain radiation question was reworded to improve clarity (35). We consider misclassification of neuropathic pain location in our study to be unlikely, as all symptom‐related questions in the baseline survey specifically referred to the big toe joint. However, it is possible that some participants misunderstood the pain radiation question, since some nonadjacent radiation patterns were reported. Third, we did not perform any quantitative sensory testing, which would have provided greater insights into the contribution of central sensitization (42).

In conclusion, in this analysis of data from a randomized trial of individuals with first MTP joint OA, 1 in 3 individuals reported symptoms suggestive of neuropathic pain. Those with possible or likely neuropathic pain were older, had worse general physical health, worse foot health, and greater pain severity at rest. Screening for neuropathic pain may be helpful in the optimum selection of interventions in clinical practice and may be worthy of consideration when designing clinical trials.

## AUTHOR CONTRIBUTIONS

All authors were involved in drafting the article or revising it critically for important intellectual content, and all authors approved the final version to be submitted for publication. Dr. Menz had full access to all of the data in the study and takes responsibility for the integrity of the data and the accuracy of the data analysis.

### Study conception and design

Menz, Landorf, Cicuttini, Roddy, Munteanu.

### Acquisition of data

Allan, Buldt.

### Analysis and interpretation of data

Menz, Landorf, Cicuttini, Roddy, Munteanu.

## Supporting information


Disclosure Form
